# Obturator Manufacturing for Oronasal Fistula after Cleft Palate Repair: A Review from Handicraft to the Application of Digital Techniques

**DOI:** 10.3390/jfb13040251

**Published:** 2022-11-17

**Authors:** Jiali Chen, Renjie Yang, Bing Shi, Yichen Xu, Hanyao Huang

**Affiliations:** 1 State Key Laboratory of Oral Diseases & National Clinical Research Center for Oral Diseases, Department of Oral and Maxillofacial Surgery, West China Hospital of Stomatology, Sichuan University, Chengdu 610041, China; 2 State Key Laboratory of Oral Diseases & National Clinical Research Center for Oral Diseases, Eastern Clinic, West China Hospital of Stomatology, Sichuan University, Chengdu 610051, China; 3 State Key Laboratory of Oral Diseases & National Clinical Research Center for Oral Diseases, Department of Oral Prosthodontics, West China Hospital of Stomatology, Sichuan University, Chengdu 610041, China

**Keywords:** additive manufacturing, oronasal fistula, palatoplasty, 3D printing

## Abstract

An oronasal fistula (ONF) is an abnormal structure between the oral and nasal cavities, which is a common complication of cleft palate repair due to the failure of wound healing. When some patients with ONF are unsuitable for secondary surgical repair, the obturator treatment becomes a potential method. The objectives of the obturator treatment should be summarized as filling the ONF comfortably and cosmetically restoring the dentition with partial function. The anatomy of patients with cleft palate is complex, which may lead to a more complex structure of the ONF. Thus, the manufacturing process of the obturator for these patients is more difficult. For performing the design and fabrication process rapidly and precisely, digital techniques can help, but limitations still exist. In this review, literature searches were conducted through Medline via PubMed, Wiley Online Library, Science Direct, and Web of Science, and 122 articles were selected. The purpose of this review was to introduce the development of the obturator for treating patients with ONF after cleft palate repair, from the initial achievement of the obstruction of the ONF to later problems such as fixation, velopharyngeal insufficiency, and infection, as well as the application of digital technologies in obturator manufacturing.

## 1. Introduction

Cleft palate is a common congenital anomaly of the oral cavity, resulting from no fusion or partial fusion of the lateral palatal eminences and nasal septum during the period of embryonic development [1], and it occurs unilaterally or bilaterally accompanied by cleft lip [2], ranging in frequency from 0.125% to 0.167% [3]. Typical symptoms include a palatal defect, inability to suck, swallowing difficulties, abnormal articulation, and severe malocclusion [3,4]. Possible causes include inheritance [1,2,5], nutrition [6], drugs [7,8], and tobacco and alcohol [3]. Surgical treatment is the first choice for cleft palate [3]. However, oronasal fistula (ONF) can happen as a complication after cleft palate repair [9,10].

An ONF is an abnormal communication between the oral and nasal cavities, clinically manifesting as a defect ranging from the alveolar process to the uvula. In addition to arising as a complication of cleft palate repair [9,10], it can also occur due to trauma, tumors, infections, and many other factors [11,12,13,14,15]. The rate of postoperative ONF ranges from 2.4% to 55%, related to the cleft width, Veau type, and surgical method [16,17,18,19,20]. ONF can result in nasal leakage, speech disorders, impaired hearing, and food reflux, significantly impacting the patient’s quality of life [21,22]. Surgical treatment is the first choice for palatal fistula repair, but for large ONF (L-ONF) it may be unavailable for some patients due to the factors including the operation, such as age and cost [21,23]. The recurrence rate after ONF repair is also high, ranging from 0% to 43% [16,19,20]. The limitations of surgical conditions and the high incidence of morbidity and recurrence of ONF make the obturator an attractive option.

The ONF obturator should fill the ONF comfortably, be stabilized in position, and not obstruct speech function. Meanwhile, the design and fabrication process of the ONF obturator should be rapid and precise. In patients with cleft palate, the structure of ONF can be complex, e.g., in those patients with bilateral complete cleft palate, when ONF occurs, the front lesion can include the lateral hard palatines and the alveolar process near the cleft, along with the isolated premaxillary space with deformed and displaced incisors, making the design and fabrication of the obturator difficult [24,25,26]. Thus, the manufacturing process of an obturator for ONF in patients with cleft palate should be more intricate.

The history of a technique’s development can reveal the thinking for solving problems, as with any medical problem, and the old problem may recur when a new technique is developed. In this review, we aimed to demonstrate the development of an obturator for treating patients with ONF after cleft palate repair, as well as to discuss the associated problems along with the development and their solutions. Meanwhile, with the development of digital technology in dentistry, new strategies—such as intraoral scanning and additive manufacturing—have been applied to improve the fabrication of dental prostheses [27,28]; thus, we also introduce the application of these techniques in the field of obturator manufacturing for patients with ONF after cleft palate repair.

## 2. Materials and Methods

A literature search was conducted through Medline via PubMed, Wiley Online Library, Science Direct, and Web of Science. Only articles in the English language were considered. A total of 314 articles were found, but 187 were not related to our purpose, so they were excluded. Abstracts, short communications, and company literature were also excluded. Finally, 127 articles were found to be relevant to this review.

In the “Development of ONF obturator” section, because we intended to explore the history and the development of oronasal fistula, we selected the publications from 1953 to 2022 using the following keywords: oronasal fistula, cleft palate, oronasal fistula obturator, and fabrication of obturator. Based on evidence from the currently available literature, Ackerman was the first to report the oronasal obturator in 1953 [29]. We selected an additional 95 articles from that point onwards. With the development of technology, a new method of fabricating obturators emerged recently [21].

In the “Digital process of ONF obturator” section, 32 articles were selected to explore the problem of the acquisition of accurate 3D images, precision fabrication of ONF obturators, and materials of ONF obturators using the following keywords: CBCT, 3D printing, oronasal fistula obturator, and material. We selected articles that introduced the process of ONF obturator fabrication clearly and in detail to explain a digital ONF obturator and the steps needed to fabricate it.

## 3. Development of ONF Obturator

### 3.1. Achievement of ONF Obstruction with the Obturator

In 1953, Ackerman introduced a maxillofacial prosthesis, which laid the foundation for expertise in this field [29]; two years later, the use of maxillofacial prostheses after cancer surgery was presented [30]. In the subsequent decades, maxillofacial prostheses were applied to patients with maxillary defects [31], oral and maxillofacial cancer patients [32], and patients after maxillectomy [33].

**Figure 1:**
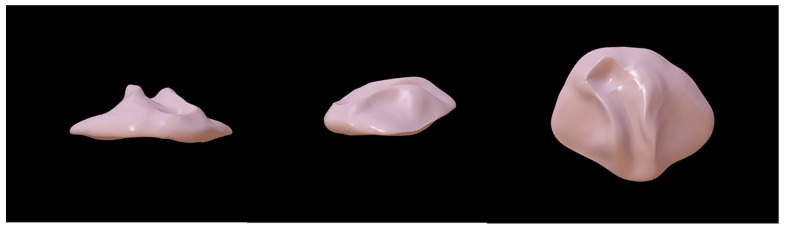
The original oronasal fistula obturator model [34]. The original oronasal fistula obturator was fabricated from hard and soft acrylics. Soft acrylic was applied on the nasal side, while self-curing hard acrylic was used to cover the soft acrylic.

In 1984, Jacobson and Rosenstein formally reported the use of an obturator in newborn patients with cleft palate, along with its manufacturing process [34]. Firstly, they obtained a model of the patient’s maxillary situation using trays and alginate impression materials, after which a plaster working model was made. Based on this plaster working model, the obturator was designed and fabricated using hard and soft acrylics. Soft acrylic was applied on the nasal side to be more comfortable, while self-curing hard acrylic was used to cover the soft acrylic in the lateral maxillary segment and the medial region (Figure 1).

### 3.2. Improvement for More Stable Retention of the Obturator

As the use of ONF obturators has increased, the placement of the obturator has been found to be essential in the outcome of the repair. In 1967, Pielou et al. used an obturator for prosthetic treatment in patients suffering from Pierre Robin syndrome [35]. To solve the problem of unstable retention, the front end of the obturator was extended to the outside of the oral cavity to form a wing-like shape, which was designed to attach to the tape. The distal end of the obturator was extended into the epiglottis, which relieved the symptom of retroflexion of the tongue back into the pharynx. However, there were other problems with this design, including the failure to achieve perfect retention when the patients had excessive oral mobility, as well as the possibility of causing other problems due to the persistent opening of the oral cavity.

**Figure 2:**
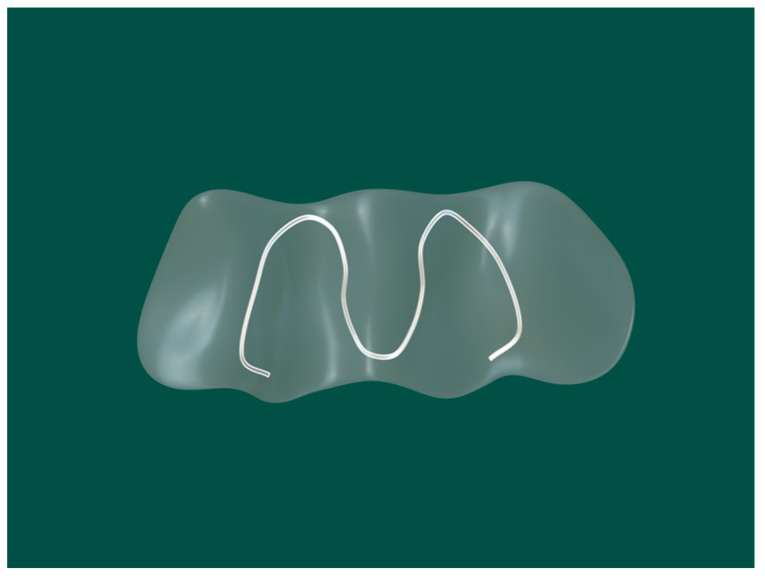
The “U”-shaped spring oronasal fistula obturator model [36]. The central part was made of silicone, and a spring was used to adjust the retention of the obstruction according to the size of the oronasal fistula.

Sullivan reported an adjustable “U”-shaped spring ONF obturator to address this problem in 1990, consisting of two components connected by a “U”-shaped spring that could be adjusted to place the obturator in the correct position (Figure 2) [36]. They also considered that the repair of ONF in infancy was a gradual process, with various treatment modalities being used as the fistula decreased until its closure [36]. During that time, the ONF obturator needed to be updated at different stages of treatment. This adjustable ONF obturator also eliminated the need for constant updating [36]. However, the long-term effect of the ONF obturator should be studied further. In patients with cleft palate, the anatomical structure is sometimes extremely complex, as no suitable fixation can be found; this merits more attention to improve obturator retention.

**Figure 3:**
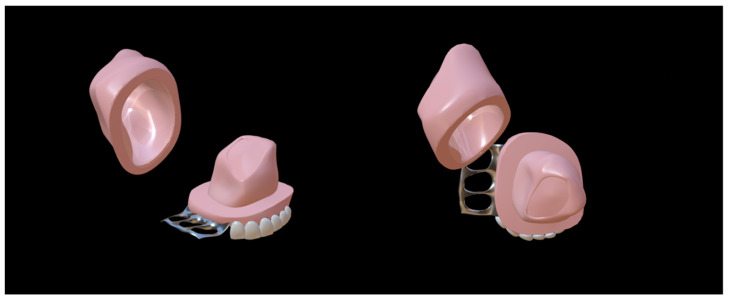
Two-piece oronasal fistula obturator with silicone bulb [41]. The silicone cap was placed over the maxillary defect, and the other part was inserted into the silicone cap.

**Figure 4:**
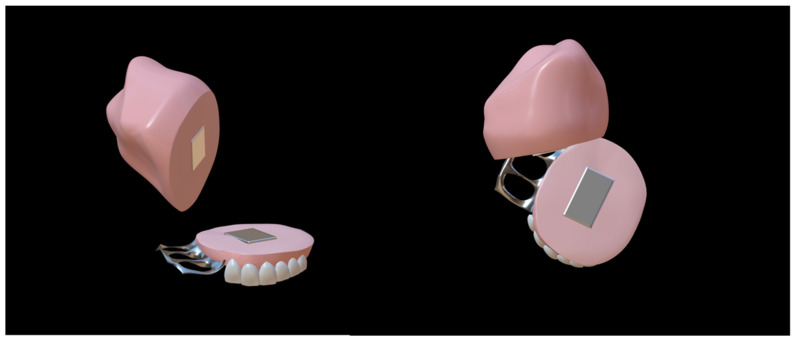
Two-piece oronasal fistula obturator model with embedded magnets [41]. The two parts of the obturator were joined together by magnets.

**Figure 5:**
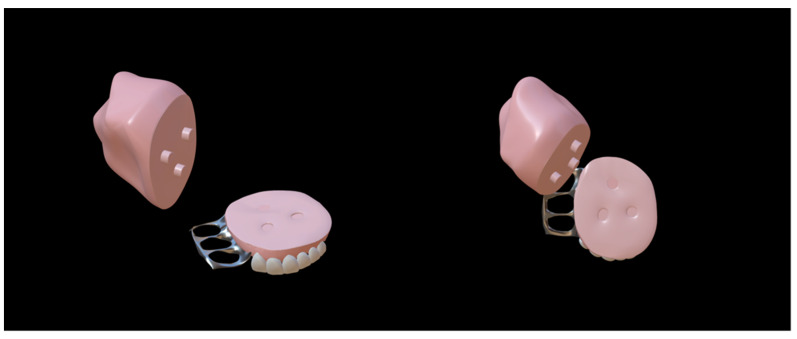
Two-piece oronasal fistula obturator model with indenters [41]. The two parts of the obturator were combined via a plug and a hole corresponding to one another.

In recent years, two-piece, claspless, and implant-fixed ONF obturators have become hotspots for the shared goal of achieving better retention. There are three main types of two-piece ONF obturator, including ONF obturators with silicone bulbs (Figure 3), ONF obturators with embedded magnets (Figure 4), and ONF obturators with indenters (Figure 5) [37]. The two-piece obturator solves the problem of the obturator’s insertion and removal, providing good comfort to the patients. The obturator without clasp fixation, as reported by Murakami et al. in 2020, consists of two parts: a palatal plate and a hollow obstruction, made of cold-curing resin using a compressed vacuum injection unit [38]. After the resin had cured, the palatal plate and the obstruction were attached with five magnetic attachments. To completely close the fistula, the rim of the obstruction was partially extended so that its posterior edge was in contact with the oral side of the soft palate at rest, and a silicone-type soft lining material was chosen for its durability. Studies have demonstrated that this innovative design increased the retention of the ONF obturator and was influential in solving food debris buildup and nasal reflux [38,39]. The implant-fixed ONF obturator designed by Buurman et al. also greatly improved retention due to the placement of the implant [40]. However, these new ONF obturators have been studied with small sample sizes and short follow-up times, and there is insufficient theoretical evidence and clinical data to prove their effectiveness—especially in patients with ONF after cleft palate repair.

### 3.3. Restoration of Speech Problems with the Obturator

The primary purpose of the ONF obturator is to obstruct abnormal communication between the oral and nasal cavities. However, in patients with cleft palate, velopharyngeal insufficiency (VPI) can also be the cause of speech problems and affect their quality of life [42,43,44,45,46,47,48,49]. Both ONF and VPI can contribute to the speech dysfunction of the patient [49,50,51], so both aspects should be considered during the manufacture of the obturator.

**Figure 6:**
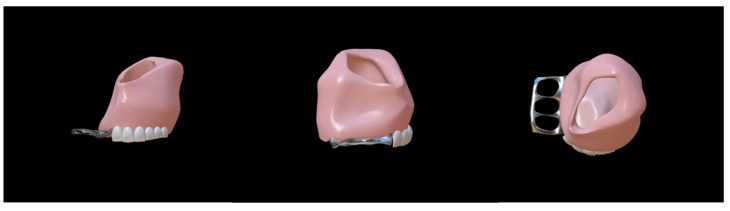
Hollow obturator model [53]. The obturator was designed to be hollow to reduce its weight and provide good retention.

In patients without VPI—such as patients after tumor excision—when the obturator is tightly integrated with the mucosa (or implanted flap), speech and swallowing functions can be restored [52]. Initially, to improve the retention of the obturator by reducing its weight, obturators were designed to be hollow, which enabled them to engage the remaining tooth and tissue bearers and extend into the defect effectively (Figure 6). Different methods have been invented, including the technique of hollowing and rejoining directly [53], the salt-losing technique [54], and the dual-processing technique [55]. In the application of hollow obturators, an interesting finding was that the design of the hollow cavity aided speech resonance, increased speech intelligibility, and gradually improved speech function [53]. Since then, numerous studies have been focused on this aspect [56,57]. An article described the clinical and laboratory procedures of a hollow bulb obturator that was used in a hemimaxillectomy patient, demonstrating that it aided speech resonance [57].

VPI can occur after the insufficient elevation of the soft palate to the pharyngeal wall, which remains as a port allowing airflow leakage [58,59,60]. After obstructing the ONF, the VPI problem remains to be solved. Blakeley was the first to use a speech bulb to improve velopharyngeal closure in a patient with cleft palate [61]. In subsequent years, studies focused on how the size and position of the speech bulb improved dysphonia [62,63,64]. However, the use of speech bulb obturators in the treatment of VPI became popular in the 20th century, partially due to techniques that permitted direct visualization of the velopharyngeal mechanism. In 1979, a palatal lift prosthesis was used to treat palatopharyngeal incompetence [65], and it was suggested that this was an effective method of improving articulation. However, this method was indicated for selected patients who have anatomically normal palates that are dysfunctional.

**Figure 7:**
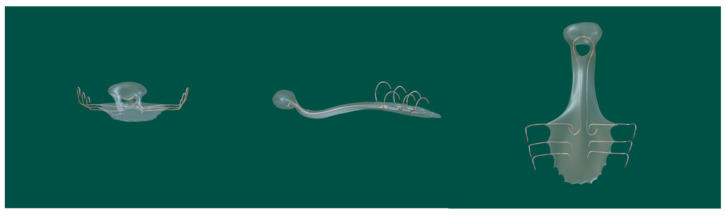
Speech bulb obturator model [67]. The obturator consisted of an acrylic front part with a fixed clip, a pharyngeal bulb, and a middle part connecting these two parts. The speech bulb improved speech resonance.

The speech bulb obturator was reported in 1993 [66], consisting of a custom-made dental appliance with an extension and advocated for use in individuals with severe pharyngeal articulation problems. Studies on speech bulb reduction later became popular once more. A case of speech function enhancement using a speech bulb was reported in detail by Bispo et al. [67]. The patient in this case report also had speech training before wearing a speech bulb obturator, but the recovery of speech function was poor. After the consultation, the authors created a removable obturator consisting of an acrylic front part with a fixed clip, a pharyngeal bulb part based on the shape of the palatopharyngeal gap, and a middle part connecting these two parts (Figure 7). During the treatment, they gradually reduced the size of the speech bulb and trained the patient, eventually improving the patient’s speech function. This case suggested that a speech bulb obturator could significantly improve the patient’s speech disorder. In addition, Elangovan [68], Fen-Huey Lin [69], Agrawal [70], and others have also reported good therapeutic outcomes with speech bulbs in ONF restoration.

### 3.4. Resolution of Infections

Infections related to implanted devices are mainly caused by *Staphylococcus* spp., especially *S. epidermidis* and *S. aureus* [71,72,73]. The incidence of fungal infections related to implanted devices is lower but more severe, most commonly caused by pathogenic *Candida* species—especially *C. albicans* [74,75,76]. Furthermore, moving the obturator prostheses frequently increases the risk of infection because they may traumatize the oral mucosa [77]. Silicones and acrylic resins are the most commonly used materials at present [78,79].

In a study by Wieckiewicz et al. [80], *Candida* adhered well to silicone, and *Candida* on obturator prostheses made of silicone and on oral mucosa was found to be the leading cause of inflammation in patients after tumor resection. In 2009, Mattos et al. [81] reported that patients using acrylic ONF obturator frequently developed stomatitis, and found that the oral mucosa under the obturator was more susceptible to *Candida albicans* infections. Higher silicone porosity and a reduced degree of acrylic polymerization have been reported to contribute to the colonization of microorganisms, leading to the development of infections [82,83,84]. These reports suggest that the choice of material is also crucial for effective antimicrobial resistance and for preventing postoperative infections and complications.

The growth of fungi has been shown to destroy the lining surface, leading to irritation of the oral tissues. Possible causes include increased surface roughness and high levels of secreted enzymes and metabolic products produced by fungal cells [85,86]. Batches of methods were tried to avoid and reduce the adhesion of these microorganisms so as to reduce related infections, including the addition of antifungal agents or antiseptics in materials, the modification of surface physicochemical characteristics, and the use of different materials.

The method of decreasing biofilm formation by incorporating various antimicrobial materials into the obturator has been the focus of many studies [87,88,89]. In 2012, Jingwei He et al. incorporated quaternary ammonium salts into methyl methacrylate (MMA) to form a quaternary ammonium methacrylate that maintained its antibacterial activity without sacrificing its mechanical properties [90]. Other attempts were made to modify the surface characteristics of the materials, including electronegativity, wettability, and roughness, so as to reduce microorganism adhesion [91,92,93]. Nikawa introduced a thermocycling process to a fabricated maxillofacial prosthesis in 2001 and observed *Candida albicans* growing on it [91]. The results suggested that the materials exhibited antifungal effects because the surface of these materials was made hydrophobic by this method. In 2007, Khalaf modified silicone elastomer surfaces with different surface roughness and porosity and concluded that a smoother, less porous surface exhibited a lower adhesion of microorganisms [93]. It was reported that parylene coating reduced the adhesion and aggregation of *C*. *Albicans* on the surface of silicone and improved the wettability of the silicone [92], while titanium offered good biomechanical properties, low weight, and high corrosion resistance, and bacteria were not able to penetrate the surface [94]. The titanium surface was also polished so that the microorganisms could not adhere easily. In one study, nanostructured materials were reported to show a slight decrease in microorganism adhesion [95].

## 4. Digital Process of ONF Obturator

Digital ONF obturators have become a new treatment modality, shortening the production process and making the ONF obturators more precise, allowing for personalized treatment. The digital ONF obturator is a 3D-printed obturator based on a model obtained via intraoral scan, CBCT technology, or other imaging techniques.

### 4.1. Acquisition of Accurate 3D Images

Advances in radiological imaging technology have facilitated the creation of 3D imaging methods, with CT being the first technique to present stereoscopic hard and soft tissues of maxilla through the acquisition capability of multiple consecutive cross-sectional images [96]. Dental cone-beam CT (CBCT) was born and became a widely used imaging tool in dental diagnostic treatment due to the changing demand [97]. Kuijpers et al. [98], in a systematic review, stated that CT, CBCT, MRI, stereophotogrammetry, and laser surface scanning were the 3D techniques most commonly used for patients with cleft lip and palate—mainly for soft tissue analysis, bone graft evaluation, and craniofacial skeleton changes. CBCT has the advantages of high spatial resolution, low radiation dose, small size, and low cost compared with conventional medical CT. A digital ONF obturator takes advantage of its ability to be reconstructed to provide a 3D view of the patient’s oral cavity. Many studies have focused on the precision of CBCT [99,100], which is the basis of the digital manufacturing [101]. Because its low-density resolution, poor soft-tissue imaging, and metal artifacts hamper the achievement of accurate intraoral images, MRI is required to obtain clear soft-tissue images to compensate for nasal-side CBCT images [102]. A patient-specific low-cost ONF obturator was explored by Bartellas et al. [27]. A CT scan of the patient’s maxilla was performed, which was visualized and rendered using OsiriX Lite after creating a model of the maxilla and then using Meshmixer to design the ONF obturator.

### 4.2. Precision Fabrication of ONF Obturators

CBCT can obtain realistic and accurate 3D oral images to assess ONF before repair. Three-dimensional (3D) printing technology can accurately print a model of the patient’s palate and a computer-designed ONF obturator. Choi et al. [103] used the 3D scanning procedure of CBCT to create 3D digital images of the patient’s palate, which were then exported to a computer for 3D analysis in a standard language format of surface subdivision. Following this analysis, they created a model of the patient’s palate using 3D printing and, by measuring it, they concluded that this technique could accurately simulate the patient’s palate condition. A detailed step-by-step description of how to design an obturator using dental CAD software (exocad DentalCAD 3.0) and produce the obturator using 3D printing was given by Krämer Fernandez et al. [104], but they stated that this method was limited to small defects. CAD/CAM systems have also been developed to manufacture fixed and removable obturators [105,106]. In 2000, Bibb [107] reported using CAD/CAM technology to fabricate prostheses and stated that CAD could be applied to produce accurate physical models based on careful acquisition of 3D scan data.

### 4.3. Further Improvements in Materials of ONF Obturators

Materials for dental treatment are also constantly being updated thanks to the advent of 3D printing. In particular, titanium and its alloys are suitable for 3D printing technology. The titanium created by 3D printing has high yield strength, ultimate strength, excellent ductility, and low solubility, which could resolve safety issues caused by the dissolution of metal ions [108]. Studies on obturator materials have been stimulated by the increasing demand for high esthetic restoration [109,110,111]. Recently, Schonhoff et al. [111] reported the mechanical properties of thermoplastic polymer materials by 3D printing, and the results showed that the mechanical properties were affected due to 3D printing, while the 3D printing parameters employed for the additive manufacturing of thermoplastic polymer material specimens require further optimization.

We also found that the following materials can potentially be used: polymethylmethacrylate (PMMA) is used widely in obturators due to its low density, aesthetics, cost-effectiveness, and stability [112,113]. In addition to mechanical properties, the material’s biocompatibility is also an important factor to consider, representing the most important biological property [114]. Studies have demonstrated that PMMA has lower toxicity [114,115]. Properly cured PMMA materials have good biocompatibility due to low amounts of monomers, such as in heat-cured and microwave-cured PMMA [116]. As mentioned above, titanium and titanium alloys are currently commonly used as 3D-printing materials in the production of ONF obturators. The titanium alloys applied in 3D printing have good biocompatibility [117]. Titanium and its alloys have good mechanical properties, corrosion resistance, and a high strength-to-weight ratio [118,119,120], so they are widely used, including in the manufacture of ONF obturators. Meanwhile, other biodegradable metal materials such as Zn-Cu-Fe alloy can also be promising [121].

Improving the antimicrobial properties of 3D-printed materials is also a current research topic that can help to reduce bacterial adhesion and inflammation after wearing ONF obturator. However, although few antimicrobial materials have been used for ONF obturators to date, we can get a glimpse of other antimicrobial dental materials for 3D printing. In the study by Chen et al. [122], the authors demonstrated that the composite resin added TiO_2_ showed good antibacterial properties compared to pure PMMA resin. Herrmann and Ren et al. [123] reported a 3D-printed polymeric resin containing antimicrobial positively charged quaternary ammonium groups, and the results showed that the quaternization of the material greatly improved its antimicrobial resistance, while the mechanical properties were similar to those of other materials. Understanding the application of other antimicrobial dental materials will allow us to apply them to ONF obturator fabrication in future studies. Moreover, anti-inflammation strategies such as cationic scavenging should also be considered [124,125].

### 4.4. Fabrication of a Digital ONF Obturator

**Figure 8:**
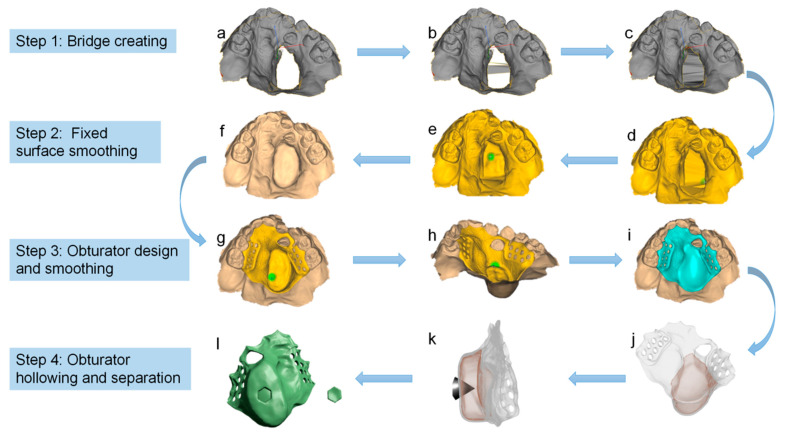
The process of manufacturing a digital ONF obturator [21] The whole process consists of four steps: bridge creation (**a**–**c**), fixed-surface smoothing (**d**–**f**), obturator design and smoothing (**g**–**i**), and hollow and separation (**j**–**l**).

Recently, the process of producing a digital ONF obturator was described in detail [21]. The first step was to obtain an intraoral situation using a confocal laser scanner, and then the probe was inserted as deep as possible to get a more complete model. Then, an ONF model was designed using CAD software, during which they first filled the ONF and then made the filled surface smooth because it could only be developed on a closed surface. Once the model had been created, the ONF obturator was designed and then hollowed out. After that, the designed ONF obturator was printed in two parts—the hexagonal anti-rotation cap and the perforated bottom, and then welded and polished. To sum up, the process of producing a digital ONF obturator is as follows (Figure 8).

## 5. Summary of the Important Designs during the Development

In 1953, Ackerman et al. reported a maxillofacial prosthesis for the first time, which laid the foundation for this field of study [29]. Then, acrylic was used in ONF obturators, and soft acrylic was used on the nasal side to increase comfort in 1984 [34]. Newly designed obturators have been used to improve the retention, including “U”-shaped [36] and two-piece ONF obturators [37]. In 2011, a hollow ONF obturator and speech ball obturator were used to aid in speech function [57,67]. Recently, digital ONF obturators have become a research hotspot; Yichen Xu et al. introduced the manufacture of ONF obturator clearly [21]. The important designs were shown in Table 1.

## 6. Summary

In patients with cleft palate, the structure of ONF is much more complex than that in patients with other problems, making the design and fabrication of obturators difficult [24,25,26]. Currently, digital ONF obturators appear to be destined to become the trend, and for patients with cleft palate their fabrication is likely to be more challenging.

There are many advantages to digital technology in the treatment of ONF, such as avoiding the inevitable errors associated with the impressions, plaster revisions, and restoration of cusp misalignments in conventional manufacturing. In addition, the ONF obturator can be fabricated in a much shorter time due to the removal of tedious steps. In the future, digital techniques will be more widely used in this field, where virtual-reality design can interact with 3D printing. Doctors may directly perform the 3D design of the restoration in the virtual world, observe the 3D restoration products to better estimate the feasibility of the products, and reduce the wastage of time and resources.

In addition to good retention and antimicrobial properties, the perfect digital ONF obturator further achieves aesthetic restoration. Today, smart biomaterials and advanced stem cell culture technology, coupled with 3D printing, provide an excellent basis for patient-tailored treatments. The 3D printing technique has the advantage that it can produce various geometries to perfectly fit any tissue defect as well as mimicking complex inner tissue architecture and heterogeneity via the precise positioning of different materials or cell types. This technology is already being used for the manufacturing of periodontal and gingival tissues [126,127], and in the future it will likely be used for the manufacturing of palatal mucosa for better functional and aesthetic restoration.

However, since acquiring accurate 3D images and 3D printing are the basis of the manufacturing process, studies on obtaining accurate 3D images and 3D printing materials should be advanced to improve the mechanical properties and reduce the gap between 3D-printed models and human structures—especially for patients with ONF after cleft palate repair. Most 3D-printed materials lack the realism to adequately mimic soft human biological tissue and its great mechanical properties, and post-processing is often required to soften the printed structure. On the other hand, materials that can be applied to 3D printing are limited. Therefore, none of the currently available materials can fully mimic elastic biological tissue, which should also be investigated in the future.

## Figures and Tables

**Table 1:** The important designs during the development.

ONF Types	Time	Materials	Highlights	Authors
Maxillofacial prosthesis	1953	——	Laid the foundation for this field	Ackerman et al. [29]
Acrylic ONF obturator	1984	Acrylic	Used soft acrylic on the nasal side to improve comfort	Jacobson et al. [34]
“U”-shaped ONF obturator	1990	Silicone and metal	Improved ONF obturator retention with spring adjustability	Sullivan et al. [36]
Two-piece ONF obturator	2015	Acrylic, silicone, magnets	Used different forms of bonding to solve the problem of obturator insertion and removal, as well as to provide good comfort to the patients	Dholam et al. [37]
Hollow ONF obturator	2011	Acrylic	The hollow design aided the speech resonance and improved the retention	Bhasin et al. [57]
Speech ball obturator	2011	Silicone	The speech ball improved the patients’ speech function	Bispo et al. [67]
Digital ONF obturator	2022	Acrylic and Ti-6Al-4V alloy	Clearly introduced the manufacturing process of a digital ONF obturator	Yichen Xu et al. [21]

## Data Availability

No applicable.

## References

[1] Paradowska-Stolarz A. (2015). MSX1 gene in the etiology orofacial deformities. Postep. Hig. Med. Dosw..

[2] Dixon M.J., Marazita M.L., Beaty T.H., Murray J.C. (2011). Cleft lip and palate: Understanding genetic and environmental influences. Nat. Rev. Genet..

[3] Vyas T., Gupta P., Kumar S., Gupta R., Gupta T., Singh H.P. (2020). Cleft of lip and palate: A review. J. Fam. Med. Prim. Care.

[4] Roguzińska S., Pelc A., Mikulewicz M. (2020). Orthodontic-care burden for patients with unilateral and bilateral cleft lip and palate. Dent. Med. Probl..

[5] Nasroen S.L., Maskoen A.M., Soedjana H., Hilmanto D., Gani B.A. (2022). IRF6 rs2235371 as a risk factor for non-syndromic cleft palate only among the Deutero-Malay race in Indonesia and its effect on the IRF6 mRNA expression level. Dent. Med. Probl..

[6] Crider K.S., Bailey L.B. (2011). Defying birth defects through diet?. Genome Med..

[7] Bateman B.T., Hernandez-Diaz S., Straub L., Zhu Y., Gray K.J., Desai R.J., Mogun H., Gautam N., Huybrechts K.F. (2021). Association of first trimester prescription opioid use with congenital malformations in the offspring: Population based cohort study. BMJ.

[8] Wilcox A.J., Lie R.T., Solvoll K., Taylor J., McConnaughey D.R., Abyholm F., Vindenes H., Vollset S.E., Drevon C.A. (2007). Folic acid supplements and risk of facial clefts: National population based case-control study. BMJ.

[9] Alonso V., Abuin A.S., Duran C., Gomez O., Miguez L., Molina M.E. (2019). Three-layered repair with a collagen membrane and a mucosal rotational flap reinforced with fibrine for palatal fistula closure in children. Int. J. Pediatr. Otorhinolaryngol..

[10] Honnebier M.B.O.M., Johnson D.S., Parsa A.A., Dorian A., Parsa F.D. (2000). Closure of Palatal Fistula with a Local Mucoperiosteal Flap Lined with Buccal Mucosal Graft. Cleft Palate-Craniofacial J..

[11] Abuabara A., Cortez A.L., Passeri L.A., de Moraes M., Moreira R.W. (2006). Evaluation of different treatments for oroantral/oronasal communications: Experience of 112 cases. Int. J. Oral Maxillofac. Surg..

[12] Ahmed M.V., Kaul D., Naz F., Tambuwala A., Chand M. (2013). Repair of iatrogenic oronasal fistula after periapical surgery. Univers. Res. J. Dent..

[13] Majid O.W. (2008). Persistent oronasal fistula after primary management of facial gunshot injuries. Br. J. Oral Maxillofac. Surg..

[14] Sahoo N.K., Desai A.P., Roy I.D., Kulkarni V. (2016). Oro-Nasal Communication. J. Craniofacial Surg..

[15] Tartaro G., Rauso R., Bux A., Santagata M., Colella G. (2008). An unusual oronasal fistula induced by prolonged cocaine snort. Case report and literature review. Minerva Stomatol..

[16] Garg R., Shah S., Uppal S., Mittal R.K. (2019). A statistical analysis of incidence, etiology, and management of palatal fistula. Natl. J. Maxillofac. Surg..

[17] Mahajan R.K., Kaur A., Singh S.M., Kumar P. (2018). A retrospective analysis of incidence and management of palatal fistula. Indian J. Plast. Surg..

[18] Yuan N., Dorafshar A.H., Follmar K.E., Pendleton C., Ferguson K., Redett R.J. (2016). Effects of Cleft Width and Veau Type on Incidence of Palatal Fistula and Velopharyngeal Insufficiency after Cleft Palate Repair. Ann. Plast. Surg..

[19] Tse R.W., Siebold B. (2018). Cleft Palate Repair: Description of an Approach, Its Evolution, and Analysis of Postoperative Fistulas. Plast. Reconstr. Surg..

[20] Shankar V.A., Snyder-Warwick A., Skolnick G.B., Woo A.S., Patel K.B. (2018). Incidence of Palatal Fistula at Time of Secondary Alveolar Cleft Reconstruction. Cleft Palate-Craniofacial J..

[21] Xu Y., Huang H., Wu M., Tian Y., Wan Q., Shi B., Hu T., Spintzyk S. (2022). Rapid Additive Manufacturing of a Superlight Obturator for Large Oronasal Fistula in Pediatric Patient. Laryngoscope.

[22] Brandão T.B., Vechiato Filho A.J., de Souza Batista V.E., de Oliveira M.C.Q., Santos-Silva A.R. (2016). Obturator prostheses versus free tissue transfers: A systematic review of the optimal approach to improving the quality of life for patients with maxillary defects. J. Prosthet. Dent..

[23] Murthy J. (2011). Descriptive study of management of palatal fistula in one hundred and ninety-four cleft individuals. Indian J. Plast. Surg..

[24] Li H., Yin N., Song T. (2015). Oronasal fistula repair using the alveolar ridge approach. Int. J. Pediatr. Otorhinolaryngol..

[25] Goiato M.C., dos Santos D.M., Moreno A., Santiago J.F.J., Haddad M.F., Pesqueira A.A., Miyahara G.I. (2011). Prosthetic Treatments for Patients with Oronasal Communication. J. Craniofacial Surg..

[26] Gümüş H.O., Tuna S.H. (2009). An alternative method for constructing an obturator prosthesis for a patient with a bilateral cleft lip and palate: A clinical report. J. Esthet. Restor. Dent..

[27] Bartellas M., Tibbo J., Angel D., Rideout A., Gillis J. (2018). Three-Dimensional Printing: A Novel Approach to the Creation of Obturator Prostheses Following Palatal Resection for Malignant Palate Tumors. J. Craniofacial Surg..

[28] Rodney J., Chicchon I. (2017). Digital Design and Fabrication of Surgical Obturators Based Only on Preoperative Computed Tomography Data. Int. J. Prosthodont..

[29] Ackerman A.J. (1953). Maxillofacial prosthesis. Oral Surg. Oral Med. Oral Pathol..

[30] Ackerman A.J. (1955). The prosthetic management of oral and facial defects following cancer surgery. J. Prosthet. Dent..

[31] Boucher L.J., Heupel E.M. (1966). Prosthetic restoration of a maxilla and associated structures. J. Prosthet. Dent..

[32] Curtis T.A. (1967). Treatment planning for intraoral maxillofacial prosthetics for cancer patients. J. Prosthet. Dent..

[33] Zarb G.A. (1967). The maxillary resection and its prosthetic replacement. J. Prosthet. Dent..

[34] Jacobson B.N., Rosenstein S.W. (1984). Early maxillary orthopedics for the newborn cleft lip and palate patient. An impression and an appliance. Angle Orthod..

[35] Pielou W.D. (1967). Non-surgical management of Pierre Robin syndrome. Arch. Dis. Child..

[36] Sullivan P.G. (1990). Early Pre-Surgical Treatment of the Cleft Palate Patient. J. R. Soc. Med..

[37] Dholam K.P., Sadashiva K.M., Bhirangi P.P. (2015). Rehabilitation of large maxillary defect with two-piece maxillary obturators. J. Cancer Res. Ther..

[38] Murakami M., Nishi Y., Shimizu T., Nishimura M. (2020). A retainer-free obturator prosthesis in a fully dentulous patient with palatal defects. J. Oral Sci..

[39] Białożyt-Bujak E., Wyszyńska M., Chladek G., Czelakowska A., Gala A., Orczykowska M., Białożyt A., Kasperski J., Skucha-Nowak M. (2021). Analysis of the Hardness of Soft Relining Materials for Removable Dentures. Int. J. Environ. Res. Public Health.

[40] Buurman D.J.M., Speksnijder C.M., Engelen B.H.B.T., Kessler P. (2020). Masticatory performance and oral health-related quality of life in edentulous maxillectomy patients: A cross-sectional study to compare implant-supported obturators and conventional obturators. Clin. Oral Implant. Res..

[41] Ayad T., Xie L. (2015). Facial artery musculomucosal flap in head and neck reconstruction: A systematic review. Head Neck.

[42] Sakran K.A., Wu M., Alkebsi K., Mashrah M.A., Al-Rokhami R.K., Wang Y., Mohamed A.A., Elayah S.A., Al-Sharani H.M., Huang H. (2022). The Sommerlad-Furlow Modified Palatoplasty Technique: Postoperative Complications and Implicating Factors. Laryngoscope.

[43] Chen N., Shi B., Huang H. (2022). Velopharyngeal Inadequacy-Related Quality of Life Assessment: The Instrument Development and Application Review. Front. Surg..

[44] Xie Z., Yang C., Zhao Y., Yang Y., Xia W., Zong Y., Chi T., Shi B., Huang H., Gong C. (2022). Anxiety in Chinese Patients with Cleft Lip and/or Palate: A Preliminary Study. Front. Pediatr..

[45] Sakran K.A., Al-Rokhami R.K., Wu M., Chen N., Yin H., Guo C., Wang Y., Alkebsi K., Abotaleb B.M., Mohamed A.A. (2022). Correlation of the Chinese velopharyngeal insufficiency-related quality of life instrument and speech in subjects with cleft palate. Laryngoscope Investig. Otolaryngol..

[46] Huang H., Chen N., Yin H., Skirko J.R., Guo C., Ha P., Li J., Shi B. (2019). Validation of the Chinese Velopharyngeal Insufficiency Effects on Life Outcomes Instrument. Laryngoscope.

[47] Ysunza P.A., Repetto G.M., Pamplona M.C., Calderon J.F., Shaheen K., Chaiyasate K., Rontal M. (2015). Current Controversies in Diagnosis and Management of Cleft Palate and Velopharyngeal Insufficiency. Biomed. Res. Int..

[48] Wang X., Guo C.L., Shi B., Yin H. (2020). Velopharyngeal closure pattern and speech characteristics of patients congenital velopharyngeal insufficiency. Hua Xi Kou Qiang Yi Xue Za Zhi.

[49] Tache A., Maryn Y., Mommaerts M.Y. (2021). Need for velopharyngeal surgery after primary palatoplasty in cleft patients. A retrospective cohort study and review of literature. Ann. Med. Surg..

[50] Smyth A.G., Wu J. (2019). Cleft Palate Outcomes and Prognostic Impact of Palatal Fistula on Subsequent Velopharyngeal Function—A Retrospective Cohort Study. Cleft Palate-Craniofacial J..

[51] Gustafsson C., Heliövaara A., Leikola J. (2021). Long-Term Follow-up of Unilateral Cleft lip and Palate: Incidence of Speech-Correcting Surgeries and Fistula Formation. Cleft Palate-Craniofacial J..

[52] Narayanraopeta S., Vemisetty H.K., Marri T., Konda P. (2020). Rehabilitation of a Unilateral Cleft Palate with Endosseous Implants in an Edentulous Elderly Patient. Contemp. Clin. Dent..

[53] Bhat A.M. (2007). Prosthetic rehabilitation of a completely edentulous patient with palatal insufficiency. Indian J. Dent. Res..

[54] Vamsi Krishna C.H., Babu J.K., Fathima T., Reddy G.V.K. (2014). Fabrication of a hollow bulb prosthesis for the rehabilitation of an acquired total maxillectomy defect. Case Rep..

[55] Oh W.S., Roumanas E.D. (2007). Optimization of Maxillary Obturator Thickness Using a Double-Processing Technique. J. Prosthodont..

[56] Bagis B., Aydoğan E., Hasanreisoğlu U. (2008). Rehabilitation of a congenital palatal defect with a modified technique: A case report. Cases J..

[57] Bhasin A.S., Singh V., Mantri S.S. (2011). Rehabilitation of Patient with Acquired Maxillary Defect, using a Closed Hollow Bulb Obturator. Indian J. Palliat. Care.

[58] Johns D.F., Rohrich R.J., Awada M. (2003). Velopharyngeal Incompetence:: A Guide for Clinical Evaluation. Plast. Reconstr. Surg..

[59] Smith B., Guyette T.W. (2004). Evaluation of cleft palate speech. Clin. Plast. Surg..

[60] Woo A. (2012). Velopharyngeal dysfunction. Semin. Plast. Surg..

[61] Blakeley R.W. (1964). The complementary use of speech prostheses and pharyngeal flaps in palatal insufficiency. Cleft Palate J..

[62] Mazaheri M., Millard R.T. (1965). Changes in nasal resonance related to differences in location and dimension of speech bulbs. Cleft Palate J..

[63] Shelton R.L., Lindquist A.F., Chisum L., Arndt W.B., Youngstrom K.A., Stick S.L. (1968). Effect of prosthetic speech bulb reduction on articulation. Cleft Palate J..

[64] Shelton R.L., Lindquist A.F., Arndt W.B., Elbert M., Youngstrom K.A. (1971). Effect of speech bulb reduction on movement of the posterior wall of the pharynx and posture of the tongue. Cleft Palate J..

[65] LaVelle W.E., Hardy J.C. (1979). Palatal lift prostheses for treatment of palatopharyngeal incompetence. J. Prosthet. Dent..

[66] Israel J.M., Cook T.A., Blakeley R.W. (1993). The use of a temporary oral prosthesis to treat speech in velopharyngeal incompetence. Facial Plast. Surg..

[67] Bispo N.H.M., Whitaker M.E., Aferri H.C., Neves J.D.A., Dutka J.D.C.R., Pegoraro-Krook M.I. (2011). Speech therapy for compensatory articulations and velopharyngeal function: A case report. J. Appl. Oral Sci..

[68] Elangovan S., Loibi E. (2011). Two-piece hollow bulb obturator. Indian J. Dent. Res. Off. Publ. Indian Soc. Dent. Res..

[69] Lin F.H., Wang T.C. (2011). Prosthodontic Rehabilitation for Edentulous Patients with Palatal Defect: Report of Two Cases. J. Formos. Med. Assoc..

[70] Agrawal K.K., Singh B.P., Chand P., Patel C.B.S. (2011). Impact of delayed prosthetic treatment of velopharyngeal insufficiency on quality of life. Indian J. Dent. Res. Off. Publ. Indian Soc. Dent. Res..

[71] Mack D., Becker P., Chatterjee I., Dobinsky S., Knobloch J.K., Peters G., Rohde H., Herrmann M. (2004). Mechanisms of biofilm formation in *Staphylococcus epidermidis* and *Staphylococcus aureus*: Functional molecules, regulatory circuits, and adaptive responses. Int. J. Med. Microbiol..

[72] Mack D., Davies A.P., Harris L.G., Rohde H., Horstkotte M.A., Knobloch J.K. (2007). Microbial interactions in Staphylococcus epidermidis biofilms. Anal Bioanal. Chem..

[73] Xu L.C., Siedlecki C.A. (2012). Submicron-textured biomaterial surface reduces staphylococcal bacterial adhesion and biofilm formation. Acta Biomater..

[74] Ramage G., Martínez J.P., López-Ribot J.L. (2006). Candida biofilms on implanted biomaterials: A clinically significant problem. FEMS Yeast Res..

[75] Nett J., Andes D. (2006). Candida albicans biofilm development, modeling a host-pathogen interaction. Curr. Opin. Microbiol..

[76] Thein Z.M., Seneviratne C.J., Samaranayake Y.H., Samaranayake L.P. (2009). Community lifestyle of Candida in mixed biofilms: A mini review. Mycoses.

[77] Beumer J., Kurrasch M., Kagawa T. (1982). Prosthetic restoration of oral defects secondary to surgical removal of oral neoplasms. CDA J..

[78] Huber H., Studer S.P. (2002). Materials and techniques in maxillofacial prosthodontic rehabilitation. Oral Maxillofac. Surg. Clin..

[79] Goiato M.C., Zucolotti B.C., Mancuso D.N., dos Santos D.M., Pellizzer E.P., Verri F.R. (2010). Care and cleaning of maxillofacial prostheses. J. Craniofacial Surg..

[80] Wieckiewicz W., Baran E., Zenczak-Wiechiewicz D., Proniexicz A. (2004). Adhesion of Candida to the obturator and oral mucosa as a cause of the presence of inflammation in patients treated surgically for neoplasia. Rev. Iberoam. Micol..

[81] Mattos B.S.C., Sousa A.A.D., Magalhaes M.H.C.G.D., Andre M., Brito E Dias R. (2009). Candida albicans in patients with oronasal communication and obturator prostheses. Braz. Dent. J..

[82] Depprich R.A., Handschel J.G., Meyer U., Meissner G. (2008). Comparison of prevalence of microorganisms on titanium and silicone/polymethyl methacrylate obturators used for rehabilitation of maxillary defects. J. Prosthet. Dent..

[83] Atay A., Piskin B., Akin H., Sipahi C., Karakas A., Saracli M.A. (2013). Evaluation of Candida albicans adherence on the surface of various maxillofacial silicone materials. J. Mycol. Médicale.

[84] Zafar M.S. (2020). Prosthodontic Applications of Polymethyl Methacrylate (PMMA): An Update. Polymers.

[85] Nikawa H., Yamamoto T., Hamada T. (1995). Effect of components of resilient denture-lining materials on the growth, acid production and colonization of Candida albicans. J. Oral Rehabil..

[86] Nikawa H., Jin C., Hamada T., Makihira S., Kumagai H., Murata H. (2000). Interactions between thermal cycled resilient denture lining materials, salivary and serum pellicles and Candida albicans in vitro. Part II. Effects on fungal colonization. J. Oral Rehabil..

[87] Casemiro L.A., Martins C.H.G., Pires-De-Souza F.D.C.P., Panzeri H. (2008). Antimicrobial and mechanical properties of acrylic resins with incorporated silver-zinc zeolite—Part I. Gerodontology.

[88] Wen J., Yeh C.K., Sun Y. (2016). Functionalized Denture Resins as Drug Delivery Biomaterials to Control Fungal Biofilms. ACS Biomater. Sci. Eng..

[89] Jo J.K., El-Fiqi A., Lee J.H., Kim D.A., Kim H.W., Lee H.H. (2017). Rechargeable microbial anti-adhesive polymethyl methacrylate incorporating silver sulfadiazine-loaded mesoporous silica nanocarriers. Dent. Mater..

[90] He J., Söderling E., Vallittu P.K., Lassila L.V.J. (2013). Investigation of double bond conversion, mechanical properties, and antibacterial activity of dental resins with different alkyl chain length quaternary ammonium methacrylate monomers (QAM). J. Biomater. Sci. Polym. Ed..

[91] Nikawa H., Jin C., Hamada T., Makihira S., Polyzois G. (2001). Candida albicans growth on thermal cycled materials for maxillofacial prostheses in vitro. J. Oral Rehabil..

[92] Zhou L., Tong Z., Wu G., Feng Z., Bai S., Dong Y., Ni L., Zhao Y. (2010). Parylene coating hinders Candida albicans adhesion to silicone elastomers and denture bases resin. Arch. Oral Biol..

[93] Khalaf S., Ariffin Z., Husein A., Reza F. (2017). Surface Coating of Gypsum-Based Molds for Maxillofacial Prosthetic Silicone Elastomeric Material: Evaluating Different Microbial Adhesion. J. Prosthodont..

[94] Tschernitschek H., Borchers L., Geurtsen W. (2005). Nonalloyed titanium as a bioinert metal-a review. Quintessence Int..

[95] Perez-Jorge C., Arenas M.A., Conde A., Hernández-Lopez J.M., de Damborenea J.J., Fisher S., Hunt A.M., Esteban J., James G. (2017). Bacterial and fungal biofilm formation on anodized titanium alloys with fluorine. J. Mater. Sci. Mater. Med..

[96] Shi B., Huang H. (2020). Computational technology for nasal cartilage-related clinical research and application. Int. J. Oral Sci..

[97] Pauwels R., Araki K., Siewerdsen J.H., Thongvigitmanee S.S. (2015). Technical aspects of dental CBCT: State of the art. Dentomaxillofac. Radiol..

[98] Kuijpers M.A.R., Chiu Y.T., Nada R.M., Carels C.E.L., Fudalej P.S. (2014). Three-dimensional Imaging Methods for Quantitative Analysis of Facial Soft Tissues and Skeletal Morphology in Patients with Orofacial Clefts: A Systematic Review. PLoS ONE.

[99] Kihara H., Hatakeyama W., Komine F., Takafuji K., Takahashi T., Yokota J., Oriso K., Kondo H. (2020). Accuracy and practicality of intraoral scanner in dentistry: A literature review. J. Prosthodont. Res..

[100] An H., Langas E.E., Gill A.S. (2022). Effect of scanning speed, scanning pattern, and tip size on the accuracy of intraoral digital scans. J. Prosthet. Dent..

[101] Auškalnis L., Akulauskas M., Jegelevičius D., Simonaitis T., Rutkūnas V. (2022). Error propagation from intraoral scanning to additive manufacturing of complete-arch dentate models: An in vitro study. J. Dent..

[102] Decazes P., Hinault P., Veresezan O., Thureau S., Gouel P., Vera P. (2020). Trimodality PET/CT/MRI and Radiotherapy: A Mini-Review. Front. Oncol..

[103] Choi Y.S., Shin H.S. (2019). Preoperative Planning and Simulation in Patients With Cleft Palate Using Intraoral Three-Dimensional Scanning and Printing. J. Craniofacial Surg..

[104] Krämer Fernandez P., Kuscu E., Weise H., Engel E.M., Spintzyk S. (2022). Rapid additive manufacturing of an obturator prosthesis with the use of an intraoral scanner: A dental technique. J. Prosthet. Dent..

[105] Williams R.J., Bibb R., Eggbeer D., Collis J. (2006). Use of CAD/CAM technology to fabricate a removable partial denture framework. J. Prosthet. Dent..

[106] Kattadiyil M.T., Mursic Z., AlRumaih H., Goodacre C.J. (2014). Intraoral scanning of hard and soft tissues for partial removable dental prosthesis fabrication. J. Prosthet. Dent..

[107] Bibb R., Brown R. (2000). The application of computer aided product development techniques in medical modelling topic: Rehabilitation and prostheses. Biomed. Sci. Instrum..

[108] Tian Y., Chen C., Xu X., Wang J., Hou X., Li K., Lu X., Shi H., Lee E.-S., Jiang H.B. (2021). A Review of 3D Printing in Dentistry: Technologies, Affecting Factors, and Applications. Scanning.

[109] Della Bona A., Cantelli V., Britto V.T., Collares K.F., Stansbury J.W. (2021). 3D printing restorative materials using a stereolithographic technique: A systematic review. Dent. Mater..

[110] Park S.M., Park J.M., Kim S.K., Heo S.J., Koak J.Y. (2020). Flexural Strength of 3D-Printing Resin Materials for Provisional Fixed Dental Prostheses. Materials.

[111] Schönhoff L.M., Mayinger F., Eichberger M., Reznikova E., Stawarczyk B. (2021). 3D printing of dental restorations: Mechanical properties of thermoplastic polymer materials. J. Mech. Behav. Biomed. Mater..

[112] Ajaj-Alkordy N.M., Alsaadi M.H. (2014). Elastic modulus and flexural strength comparisons of high-impact and traditional denture base acrylic resins. Saudi Dent. J..

[113] Meng T.R., Latta M.A. (2005). Physical properties of four acrylic denture base resins. J. Contemp. Dent. Pract..

[114] Gautam R., Singh R.D., Sharma V.P., Siddhartha R., Chand P., Kumar R. (2012). Biocompatibility of polymethylmethacrylate resins used in dentistry. J. Biomed. Mater. Res. Part B Appl. Biomater..

[115] Kedjarune U., Charoenworaluk N., Koontongkaew S. (1999). Release of methyl methacrylate from heat-curved and autopolymerized resins: Cytotoxicity testing related to residual monomer. Aust. Dent. J..

[116] Raszewski Z. (2020). Influence of polymerization method on the cytotoxicity of three different denture base acrylic resins polymerized in different methods. Saudi J. Biol. Sci..

[117] Mitra I., Bose S., Dernell W.S., Dasgupta N., Eckstrand C., Herrick J., Yaszemski M.J., Goodman S.B., Bandyopadhyay A. (2021). 3D Printing in alloy design to improve biocompatibility in metallic implants. Mater. Today.

[118] Heinl P., Müller L., Körner C., Singer R.F., Müller F.A. (2008). Cellular Ti–6Al–4V structures with interconnected macro porosity for bone implants fabricated by selective electron beam melting. Acta Biomater..

[119] Zhang S., Wei Q., Cheng L., Li S., Shi Y. (2014). Effects of scan line spacing on pore characteristics and mechanical properties of porous Ti6Al4V implants fabricated by selective laser melting. Mater. Des..

[120] Bose S., Ke D., Sahasrabudhe H., Bandyopadhyay A. (2018). Additive manufacturing of biomaterials. Prog. Mater. Sci..

[121] Xu Y., Xu Y., Zhang W., Li M., Wendel H.P., Geis-Gerstorfer J., Li P., Wan G., Xu S., Hu T. (2022). Biodegradable Zn-Cu-Fe Alloy as a Promising Material for Craniomaxillofacial Implants: An in vitro Investigation into Degradation Behavior, Cytotoxicity, and Hemocompatibility. Front. Chem..

[122] Chen S.G., Yang J., Jia Y.G., Lu B., Ren L. (2019). TiO(2) and PEEK Reinforced 3D Printing PMMA Composite Resin for Dental Denture Base Applications. Nanomaterials.

[123] Yue J., Zhao P., Gerasimov J.Y., van de Lagemaat M., Grotenhuis A., Rustema-Abbing M., van der Mei H.C., Busscher H.J., Herrmann A., Ren Y. (2015). 3D-Printable Antimicrobial Composite Resins. Adv. Funct. Mater..

[124] Tu Z., Zhong Y., Hu H., Shao D., Haag R., Schirner M., Lee J., Sullenger B., Leong K.W. (2022). Design of therapeutic biomaterials to control inflammation. Nat. Rev. Mater..

[125] Huang H., Pan W., Wang Y., Kim H.S., Shao D., Huang B., Ho T.C., Lao Y.H., Quek C.H., Shi J. (2022). Nanoparticulate cell-free DNA scavenger for treating inflammatory bone loss in periodontitis. Nat. Commun..

[126] Lee V.K., Dai G. (2016). Printing of Three-Dimensional Tissue Analogs for Regenerative Medicine. Ann. Biomed. Eng..

[127] Buskermolen J.K., Reijnders C.M.A., Spiekstra S.W., Steinberg T., Kleverlaan C.J., Feilzer A.J., Bakker A.D., Gibbs S. (2016). Development of a Full-Thickness Human Gingiva Equivalent Constructed from Immortalized Keratinocytes and Fibroblasts. Tissue Eng. Part C Methods.

